# The Renoprotective Mechanisms of Sodium-Glucose Cotransporter-2 Inhibitors (SGLT2i)—A Narrative Review

**DOI:** 10.3390/ijms25137057

**Published:** 2024-06-27

**Authors:** Liana Iordan, Laura Gaita, Romulus Timar, Vlad Avram, Adrian Sturza, Bogdan Timar

**Affiliations:** 1 “Pius Brinzeu” Emergency County Hospital, 300723 Timisoara, Romania; iordanliana@gmail.com (L.I.); timar.romulus@umft.ro (R.T.); avram.vlad@umft.ro (V.A.); sturza.adrian@umft.ro (A.S.); timar.bogdan@umft.ro (B.T.); 2 Second Department of Internal Medicine, “Victor Babes” University of Medicine and Pharmacy, 300041 Timisoara, Romania; 3 Department of Functional Sciences, “Victor Babes” University of Medicine and Pharmacy, 300041 Timisoara, Romania

**Keywords:** chronic kidney disease, type 2 diabetes mellitus, renoprotection, SGLT2i, prevention

## Abstract

Chronic kidney disease (CKD) is a noncommunicable condition that has become a major healthcare burden across the globe, often underdiagnosed and associated with low awareness. The main cause that leads to the development of renal impairment is diabetes mellitus and, in contrast to other chronic complications such as retinopathy or neuropathy, it has been suggested that intensive glycemic control is not sufficient in preventing the development of diabetic kidney disease. Nevertheless, a novel class of antidiabetic agents, the sodium-glucose cotransporter-2 inhibitors (SGLT2i), have shown multiple renoprotective properties that range from metabolic and hemodynamic to direct renal effects, with a major impact on reducing the risk of occurrence and progression of CKD. Thus, this review aims to summarize current knowledge regarding the renoprotective mechanisms of SGLT2i and to offer a new perspective on this innovative class of antihyperglycemic drugs with proven pleiotropic beneficial effects that, after decades of no significant progress in the prevention and in delaying the decline of renal function, start a new era in the management of patients with CKD.

## 1. Introduction

### 1.1. Epidemiology of Type 2 Diabetes Mellitus

The past few decades have brought a significant shift in the causes of mortality and morbidity across the globe, with a continuously growing prevalence of noncommunicable diseases to the extent that they are considered to be an epidemic of the modern world [1]. One of these diseases is diabetes mellitus (DM), a progressive metabolic condition characterized by chronic hyperglycemia caused by an inadequate pancreatic β-cell secretion, an impaired action of insulin, or both, in different proportions. This condition is associated with a high burden in mortality and morbidity, a 2–4 higher risk of cardiovascular disease (CVD), and, if not controlled, with microvascular complications (diabetic retinopathy and diabetic kidney disease), an accelerated atherosclerotic macrovascular disease (coronary, cerebral, and peripheral), and diabetic neuropathy. These complications and comorbid associations are caused either directly by poor glycemic control or indirectly through other risk factors commonly identified in patients with DM such as hypertension, dyslipidemia, or obesity—especially in type 2 DM (T2DM) patients which represent approximately 90% of the total number of patients with DM—with a subsequent decrease in their life expectancy and quality of life, leading to the necessity of lifelong management that includes multifactorial risk reduction strategies beyond glycemic control [2,3,4,5]. The worldwide impact of DM is reflected in reports of the World Health Organization and the International Diabetes Federation (IDF) which estimated in 2021 a number of 537 million individuals with DM, from which almost half were undiagnosed, with a projection of an increase to 784 million individuals in 2045. Moreover, approximately 6.7 million deaths were attributed to DM in 2021, with an estimation that T2DM-related mortality will double by 2030 in the absence of a correct and comprehensive treatment [4,5,6,7,8,9,10,11,12,13,14].

### 1.2. Epidemiology of Chronic Kidney Disease

Another noncommunicable condition considered to be a major healthcare burden associated with disproportionately low awareness, chronic kidney disease (CKD), has recently witnessed a mortality-related paradox, with a continuously increasing number of deaths in absolute values attributed to this cause, especially with the increase in the severity of the disease; in the context of a lower risk of mortality in patients with end-stage kidney disease, the situation is explained by an increasing number of patients with kidney function decline—more than 800 million individuals that represent more than 10% of the general population [15,16,17,18].

### 1.3. CKD in Patients with DM

The majority of patients with CKD are diagnosed with hypertension, DM, or CVD, and since the main causes of this disease are the DM itself—diabetic kidney disease (DKD) is the main cause of kidney disease across the globe—hypertension and only after those, in order of prevalence, specific renal disorders such as glomerulonephritis. Additionally, DKD is the cause for which more than 50% of individuals enter a dialysis or kidney transplant program in the United States of America and it is noteworthy that, compared to other complications of DM, the prevalence of DKD did not decrease in the past 3 decades, irrespective of the rapid development of modern therapeutic agents used in the management of T2DM, suggesting that an intensive glycemic control is not sufficient in successfully delaying the development or progression of renal dysfunction [19].

Nevertheless, hyperglycemia is the main factor that contributes to the development of DKD, and once established, numerous pathophysiological changes such as dysfunctional tubuloglomerular feedback, renal hypoxia, lipotoxicity, podocyte injury, albuminuria, inflammation, mitochondrial dysfunction, impaired autophagy, and increased sodium-hydrogen exchanger activity contribute to a progressive decrease of the glomerular filtration rate (GFR) with glomerular sclerosis, the fundamental components of DKD. However, the proportion in which each of these abnormalities contributes to the progression of DKD remains to be established [20,21,22,23,24,25].

### 1.4. SGLT2i—An Emerging Treatment in Patients with DM

Despite the major impact of CKD and, specifically, of DKD on mortality and morbidity, recently, the therapeutic options in the management of patients with this condition have remained limited until recently when sodium-glucose cotransporter-2 inhibitors (SGLT2i) have emerged as new disease-modifying drugs [26]. SGLT2i are noninsulinic antihyperglycemic agents that act by significantly inhibiting the reabsorption of sodium and glucose in the proximal renal convoluted tubules resulting in an increased urinary excretion of glucose and a mild osmotic diuresis [27].

The glucose reabsorption via the SGLTs on the apical membrane of the proximal tubule is an active transport process that involves the Na^+^/K^+^-ATPase and then the glucose is transported on the basolateral membrane to enter the bloodstream [28,29]. The unique insulin-independent mechanism of action in which SGLT2i are blocking this process results in treating hyperglycemia while avoiding hypoglycemia, promoting weight loss, lowering blood pressure, and, more importantly, lowering the risk of major adverse cardiovascular events (MACE), with a safety profile that includes as main side effects a slight increase in the incidence of genital mycotic infections, euglycemic ketoacidosis, and, for some agents in this class, an increased risk of lower limb amputations. Nevertheless, SGLT2i have revolutionized the treatment of patients with T2DM with emerging evidence that shows their capacity for improving cardiorenal outcomes in patients with heart failure and CKD, regardless of the presence of T2DM [30,31,32,33]. This class of antidiabetic drugs has proven to reverse and counteract multiple of the pathophysiological abnormalities that lead to the development and progression of DKD and although many of these mechanisms have been clearly observed, many others remain unknown and need further investigation [34,35,36,37,38,39,40,41,42,43,44,45].

## 2. The Renal Effects of DM and the Protective Mechanisms of SGLT2i

The already discovered mechanisms through which SGLT2i contribute to renal protection can be classified as metabolic, hemodynamic, and direct renoprotective effects, although multiple other structures have been proposed by various authors. The metabolic effects, namely the general, systemic ones, include the reduction in plasma glucose levels, reduction of glucotoxicity, improvement of insulin sensitivity, reduction of body weight with the reduction of hepatic steatosis, reduction of lipotoxicity, reduction of triglycerides and small-dense LDLc, reduction of plasma uric acid levels and metabolic reprogramming with the restoration of energy efficiency by switching glucose to more energy efficient metabolites. The hemodynamic effects, largely caused by the natriuretic effects of SGLT2i, include the improvement of salt sensitivity, reduction of blood pressure, improvement of sympathetic nerve hyperactivity, reduction of volume overload, and modulation of the tubuloglomerular feedback. Lastly, the miscellaneous direct renoprotective effects include the inhibition of the Na-H exchanger, reduction of endothelial dysfunction and oxidative stress, reduction of renal hypoxia, improvement of mitochondrial dysfunction, improvement of podocyte loss, reduction of albuminuria, reduction of renal fibrosis, reduction of inflammation, improvement of erythropoiesis, reduction of tubular senescence, improvement of renal autophagy, and reduction of glomerular damage (Table 1).

Although it is evident that multiple pathogenic mechanisms lead to the development and progression of DKD, the main etiopathogenic factors are represented by hyperglycemia and hypertension and, once the condition is already installed, even a perfect glycemic and blood pressure control cannot stop its progression; it can only reduce the renal function decline rate, indicating the impact of the other pathogenic mechanisms in the development and evolution of this disease. Through all of the previously mentioned mechanisms and many undiscovered others, both indirect, related to their glucose-lowering and metabolic effects, and direct, at the renal level, independent of glucose control, SGLT2i have an important role in preventing the onset and slowing the progression of DKD, contributing significantly to achieving nephroprotection. Moreover, the metabolic and hemodynamic effects of SGLT2 inhibition contribute to the multiple cardiovascular benefits of this therapeutic class by reducing blood pressure and plasma volume, decreasing the cardiac sympathetic nervous activity, reducing body weight, and increasing the usage of ketone bodies in the myocardial cells. Nevertheless, the potential mechanisms that lead to organ protection are incompletely understood, although the SLGT2i effects in reducing the intraglomerular pressure and hyperfiltration through the inhibition of the tubuloglomerular feedback are well known as key factors, alongside the reduction of local inflammation and of mediators of maladaptive repair and fibrosis at a cellular level and the reduction of oxidative stress and the activation of mitochondrial autophagy with subsequent biogenesis. Additionally, the effects of SGLT2i in preventing kidney disease are yet to be established, although their glucose-lowering effects—without being associated with an increased risk of hypoglycemia—and their numerous renoprotective effects would benefit from studies that could contribute to a clearer image of their early actions [34,46,47,48,49].

**Figure 1:**
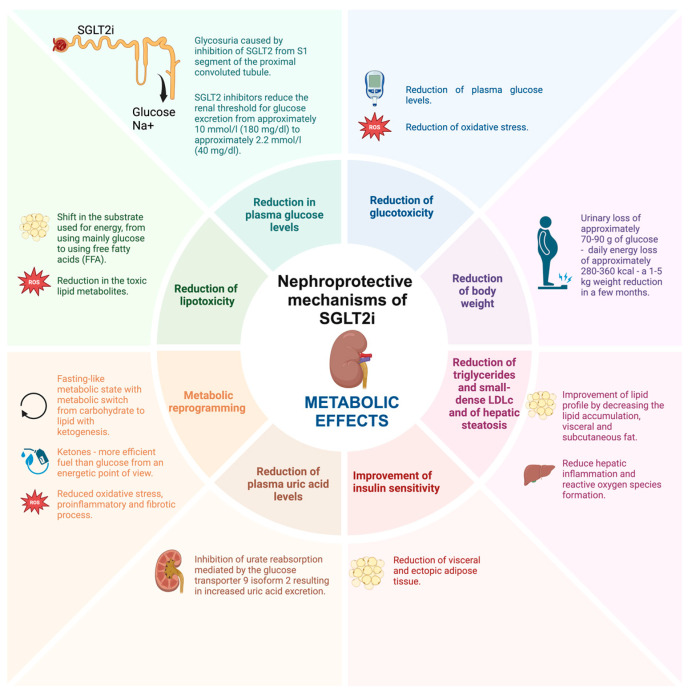
The metabolic nephroprotective mechanisms of SGLT2i (created with BioRender.com).

**Figure 2:**
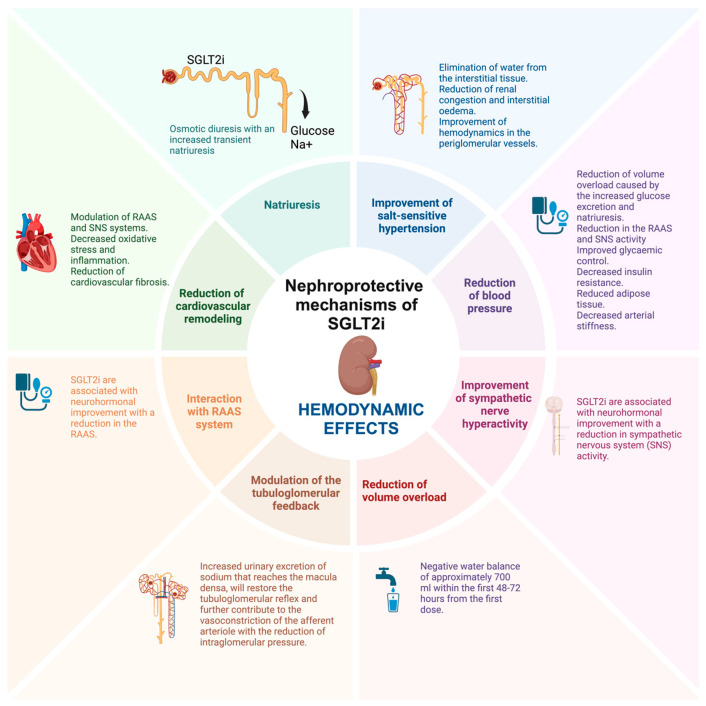
The hemodynamic nephroprotective mechanisms of SGLT2i (created with BioRender.com).

**Figure 3:**
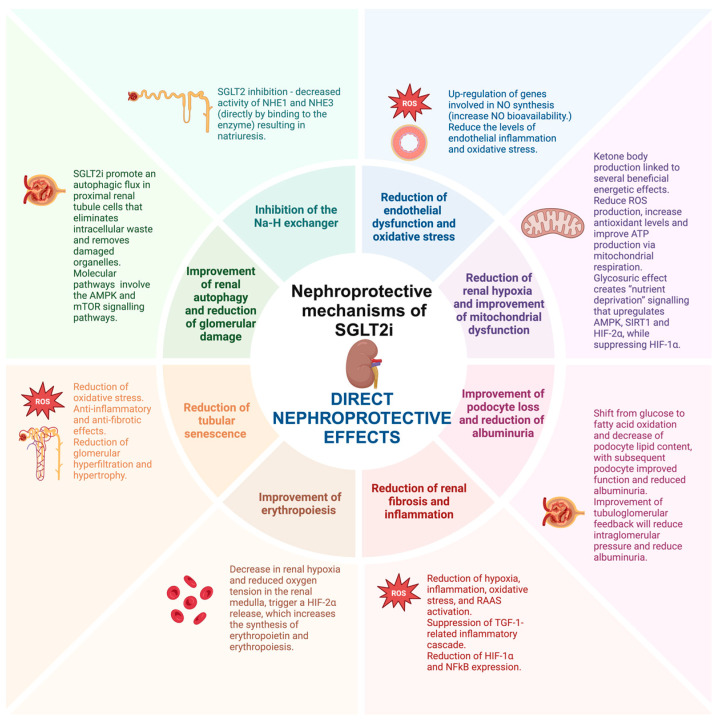
The direct nephroprotective mechanisms of SGLT2i (created with BioRender.com).

The metabolic, hemodynamic, and direct nephroprotective effects of SGLT2i are summarized in Figure 1, Figure 2 and Figure 3.

### 2.1. Metabolic Effects of SGLT2i

**Figure 4:**
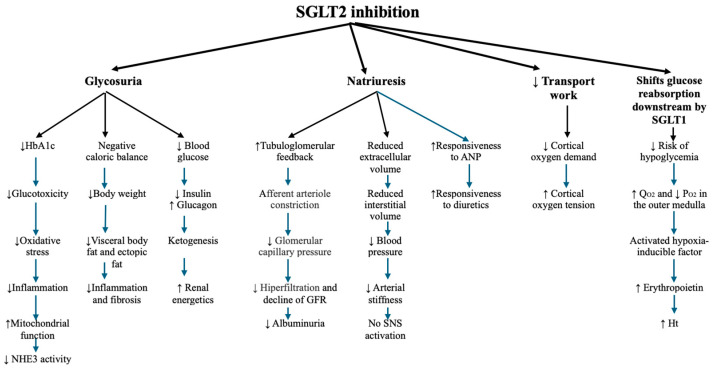
The metabolic pathways of SGLT2i (adapted from Heerspink et al. and Vallon et al.) [27,46]. ↑ increase, ↓ decrease.

The pathways through which the SGLT2i are protecting the renal function are presented in Figure 4.

#### 2.1.1. Reduction in Plasma Glucose Levels and Glucotoxicity

Hyperglycemia is the main factor that contributes to the onset of DKD—patients with a longer duration of poor glycemic control are susceptible to developing an impaired kidney function, while individuals with a normal glucose metabolism and normal levels of HbA1c do not develop this condition. Moreover, The United Kingdom Prospective Diabetes Study has demonstrated that each 1% reduction in HbA1c reduced the risk of microvascular complications by 37% and the risk of microalbuminuria by 33% in individuals with T2DM. Nevertheless, it has been shown that the normalization of mean blood glucose levels in patients who have already been diagnosed with DKD may slow its progression, without stopping it however [34].

The impact of DKD is noteworthy—most cases of CKD are cardiometabolic in nature and 42% are caused by diabetes itself—and despite advances in monitoring and treating diabetes, it remains the most common cause of end-stage kidney disease, with one in two T2D patients diagnosed with CKD in their lifetime. These outcomes are generated by the effects of hyperglycemia that contribute to increased oxidative stress and, consequently, to impaired kidney function. In patients with DM, glucose enters the cells in the renal glomerulus and tubules and is diverted into non-glycolytic pathways including the hexosamine and aldose reductase pathways, with an increase of the polyol pathway activity and subsequent oxidative stress, resulting in the glycosylation of proteins and production of advanced glycation end products, with effects of mitochondrial dysfunction, oxidative stress, and inflammation. In order to counteract these mechanisms, an early and multifactorial therapeutic approach is recommended, especially since new pharmacologic agents with pleiotropic beneficial effects have been developed, such as the SGLT2i [24,25,50,51].

This class of antihyperglycemic drugs improves fasting plasma glucose (FPG) and postprandial plasma glucose (PPG) levels and reduces HbA1c by approximately 1% (if the baseline value is between 8–8.5%) [34,52]. These effects are primarily mediated by the glycosuria that results from inhibiting the SGLT2 which are located predominantly in the S1 segment of the proximal convoluted tubule, the transporter responsible for the reabsorption of more the 90% of the filtered glucose [53]. The SGLT2 inhibitors reduce the renal threshold for glucose excretion from approximately 10 mmol/L (180 mg/dL) to approximately 2.2 mmol/L (40 mg/dL) and the resulting glycosuria can exceed 100 g in DM patients and 50–60 g in individuals with normal glucose metabolism, effects attenuated in patients with an eGFR < 50 mL/min/1.73 m^2^ and minimal when the eGFR < 30 mL/min/1.73 m^2^ [54,55]. By reducing plasma glucose levels, glucotoxicity—the toxic effect of hyperglycemia—and insulin resistance and by increasing beta-cell function, SGLT2 inhibitors reverse the previously mentioned intracellular metabolic effects, including oxidative stress, with subsequent renal protection. These indirect mechanisms will be described in the following paragraphs [7,56,57,58].

#### 2.1.2. Reduction of Body Weight

T2DM is strongly correlated with obesity, currently assessed through the value of the body mass index (BMI), although this parameter has crucial limitations caused by its inability to differentiate between adipose tissue and muscle mass and their distribution. Although more than 80% of patients with T2DM have excessive weight—either overweight or obesity—this metabolic disorder is more closely associated with the percentage of adiposity and its distribution, especially with abdominal obesity with a high percentage of visceral adipose tissue. Moreover, it has been proven that obesity itself, independently from the presence of DM, dyslipidemia, or hypertension, is associated with an increased risk of developing CKD [59,60,61].

SGLT2i contribute to a daily urinary loss of approximately 70–90 g of glucose which further generates a daily energy loss of approximately 280–360 kcal, with a result of a 1–5 kg weight reduction in a few months, according to different studies with a greater reduction noticed in patients with a higher baseline HbA1c. About 2/3 of this weight loss consists of fat mass, while the remaining 1/3 consists of lean mass; therefore, the treatment with SGLT2i improves body composition in patients with T2DM, including the values of weight itself, fat mass, BMI, waist circumference, subcutaneous fat area, and, importantly, visceral fat area. In order to conserve the muscle mass and the physical capacity—or, better, even to improve them—strategies such as structured physical activity are recommended [30,62,63,64,65,66]. It is noteworthy that even a modest weight reduction caused by the SGLT2i contributes significantly to a reduction in lipotoxicity and to an increase in the production of ketones with subsequent prevention of renal function impairment through glomerular and also tubular protective effects [47,67,68,69,70,71].

#### 2.1.3. Reduction of Lipotoxicity, Triglycerides, Small-Dense LDLc, and Insulin Sensitivity

Lipotoxicity, the ectopic accumulation of lipids in other organs than the adipose tissue, triggers the development of cell dysfunction and even cell death caused by the activation of multiple metabolic, inflammatory, and oxidative pathways. Both lipotoxicity itself and together with glucotoxicity contribute to the impairment of the beta-cell function and to the increase in insulin resistance related to muscle and hepatic cells, therefore becoming pathogenic aspects of T2DM. Moreover, atherogenic dyslipidemia, a condition characterized by elevated levels of triglycerides, small-dense low-density lipoprotein (LDL) particles, apolipoprotein B (apoB) and lipoprotein (a) (Lp(a)) with reduced levels of high-density lipoprotein (HDL) cholesterol, is commonly encountered in patients with T2DM. A similar pattern of lipid disturbances can be found in patients with diabetic nephropathy, even from its early stages, since the condition leads to several imbalances in the biosynthesis, transport, and clearance of lipids and lipoproteins. The renal lipotoxicity itself, defined by the accumulation of lipids in podocytes, mesangial cells, and proximal tubule epithelial cells, contributes to the onset of renal impairment in patients with T2DM through glomerulosclerosis, glomerular injury, and podocyte death via lipid accumulation and tubulointerstitial disease [72,73,74,75].

The SGLT2i cause a shift in the substrate used for energy, from using mainly glucose to using free fatty acids (FFAs) that induce a reduction in the toxic lipid metabolites that are found in cells, including in podocytes, mesangial cells, and proximal tubular cells, with nephroprotective effects [76]. Additionally, the reduction of visceral and ectopic adipose tissue, resulting from the treatment with SGLT2i in patients with T2DM, leads to improved insulin sensitivity, whereas the same treatment reduces, in quantity, the plasmatic levels of triglycerides and total cholesterol, with beneficial qualitative changes on the LDL and HDL particles [34,77,78].

#### 2.1.4. Reduction of Hepatic Steatosis

T2DM is not the only noncommunicable disease in which insulin resistance, lipotoxicity, oxidative stress, and inflammation have a key role. These obesity-associated mechanisms have been proven to contribute to the development of non-alcoholic fatty liver disease (NAFLD) or, the more recently defined, metabolic dysfunction-associated fatty liver disease (MAFLD) [79,80,81,82,83]. Moreover, NAFLD, especially in its necro-inflammatory form, nonalcoholic steatohepatitis (NASH), is shown to interact directly with renal function, being a marker of kidney damage and also a mechanism involved in its pathogenesis. Kidney injury could be caused by pathogenic mediators that are released from the steatotic and inflamed liver, such as reactive oxygen species, advanced glycation end products, C-reactive protein (CRP), proinflammatory, profibrogenic, and anti-fibrinolytic molecules [80,84].

In this context, the treatment with SGLT2i has been shown to prevent the progression of NAFLD with reduced hepatocellular injury, with improved transient elastography results and biomarkers such as liver enzymes [79,85,86]. Moreover, the SGLT2i have proved to reduce inflammation and reactive oxygen species formation, both key mechanisms in the pathogenesis of NAFLD and NASH [79,87,88].

#### 2.1.5. Reduction of Plasma Uric Acid Levels

Another independent risk factor for the development of DKD is hyperuricemia, a condition also associated with an increased risk of atherosclerosis and, consequently, of CVD. Moreover, the reverse is also valid since the presence of DKD, especially in more advanced stages, leads to secondary hyperuricemia [74,89].

The treatment with SGLT2i contributes to the reduction of serum uric acid levels through its increased urinary elimination; however, the mechanisms are not yet fully identified. Some of them include the inhibition of urate reabsorption mediated by the glucose transporter 9 isoform 2, resulting in increased uric acid excretion [90,91,92].

#### 2.1.6. Metabolic Reprogramming—Restoration of Energy Efficiency by Switching Glucose to More Energy-Efficient Metabolites

Metabolic alterations in the kidney that are induced by increased glycemic and lipidic levels are associated with an important role in DKD progression. The tricarboxylic acid cycle and glycolysis are accelerated in the diabetic kidney cortex and these increased pathways that are encountered in the early stages of DKD are linked to mitochondrial electron transport chain dysfunctions, leading to a less efficient ATP production and also to the progression of renal impairment [93,94,95].

SGLT2i induce a fasting-like metabolic state due to an energy and glucose deficit and will produce a metabolic switch from carbohydrate to lipid usage and ketogenesis with subsequent nutrient deprivation pathway activation and effects of reduced oxidative stress and reduced proinflammatory and fibrotic processes. These pharmacologic agents will also contribute to an increased uptake of free fatty acids in muscles and to increased catabolic pathways. Ketones, by being a more efficient fuel than glucose from an energetic point of view, contribute to lower oxygen consumption and consecutive renal protection. Additionally, the beneficial effects of SGLT2i appear to mirror the ones related to beta-aminoisobutyric acid (BAIBA), a non-proteinogenic amino acid that has been identified as a mediator of exercise-induced organ protection, which leads to the browning of white adipocytes, stimulates β-oxidation in hepatic cells, decreases body weight, and improves glycemic and lipidic control. Moreover, ketones inhibit the rapamycin complex 1 that is associated with the development of renal injury, while the stabilization of the energetical mechanisms in the tubular epithelial cells will lead to the reduction of ATP consumption and redistribution of energy to processes such as cellular repair and autophagy [34,47,94,96,97,98,99,100,101].

### 2.2. Hemodynamic Effects of SGLT2i

#### 2.2.1. Natriuresis and Improvement of Salt-Sensitive Hypertension

Patients with DM often associate renal sodium retention, and while the mechanisms are yet to be established, the increased urinary reabsorption is presumably linked to combined effects of aldosterone dysregulation, hyperglycemia, and hyperinsulinemia [102].

The mechanism of the SGLT2i includes the increased urinary glucose excretion that leads to osmotic diuresis, with an increased transient natriuresis—also caused by the reduced reabsorption in the proximal tubule—that has been proven both in animal studies and in trials that have assessed especially the urinary sodium excretion of these pharmacologic agents. Moreover, the water is removed predominantly from the interstitial tissue with consequences in reducing renal congestion and interstitial edema, without a significant impact on the intravascular volume. These effects also contribute to the improvement of hemodynamics in the periglomerular vessels and the glomeruli, to the prevention of salt-induced hypertension, and to the reduction of volume overload [42,103,104].

#### 2.2.2. Reduction of Blood Pressure, Improvement of Sympathetic Nerve Hyperactivity, and Reduction of Volume Overload

After the negative impact of increased blood glucose levels on renal function, hypertension is another major risk factor that leads to the progression of DKD, a condition frequently associated with DM, with a prevalence of over 60% in this population. It has been shown that normotensive patients with advanced renal disease have a slower progression of this condition than those with hypertension, while the activation of the renin-angiotensin-aldosterone system (RAAS) has a major involvement in the pathophysiology of DKD, with renoprotective effects being associated to the RAAS blockade [34,52,105,106,107,108].

Nevertheless, there are multiple pieces of evidence that SGLT2i are at least as effective as RAAS blockers in preventing the progression of DKD. The osmotic diuresis and reduction of volume overload caused by the increased glucose excretion and natriuresis and the consecutive negative water balance of approximately 700 mL within the first 48–72 h, from the first dose, have been hypothesized to explain the rapid blood pressure changes induced by SGLT2i with approximately 3–7/1–3 mmHg, sustained over time [52,78,109,110,111,112].

Additionally, SGLT2i are associated with neurohormonal improvement with a reduction in the RAAS and sympathetic nervous system (SNS) activity, and these alongside other beneficial effects, such as improved glycemic control, decreased insulin resistance, reduced adipose tissue, decreased arterial stiffness, and the already mentioned osmotic diuresis and natriuresis, contribute to decreased blood pressure values [113,114,115,116,117,118,119]. This reduction of blood pressure is generally observed regardless of the presence of hypertension and also in patients with lower eGFR values and is followed by a reduction in kidney disease progression, although, since its modest magnitude, other protective factors appear to play more important roles [120,121,122].

#### 2.2.3. Modulation of the Tubuloglomerular Feedback

In patients with DM, the increased activity of SGLT2 leads to an increased sodium and glucose reabsorption in the proximal convoluted tubule with a subsequently decreased level of sodium in the macula densa and will result in vasodilation of the afferent arteriole and vasoconstriction of the efferent arteriole, increased renal plasma flow, intraglomerular pressure, and hyperfiltration, which will lead to the progression of renal disease [34,46].

The SGLT2i, through increased urinary excretion of sodium that reaches the macula densa, will restore the tubuloglomerular reflex and further contribute to the vasoconstriction of the afferent arteriole with the reduction of intraglomerular pressure [46,63,123,124,125]. This reduced pressure contributes to the reduction of albuminuria and a transient drop in the eGFR rate, followed by a slower decline rate of renal function and, consecutively, with global important renoprotective effects compared to other agents [126,127]. Moreover, recent data have shown that SGLT2i do not increase vascular resistance and suggested that their beneficial effects in reducing intraglomerular pressure could be caused by postglomerular vasodilation and not mainly by a preglomerular mechanism. These previously mentioned effects can also lead to the reduction of the stress on structures such as the podocytes, with additional benefits in slowing the progression of CKD [128,129].

### 2.3. Direct Nephroprotective Effects

#### 2.3.1. Inhibition of the Na-H Exchanger

Na^+^/H^+^ exchanger (NHE) is a membrane protein that exchanges intracellular hydrogen (H^+^) ions for extracellular sodium (Na^+^) ions in 1:1 stoichiometry, a process required for the transepithelial Na^+^ transport and water absorption and for the intracellular pH regulation, essential for the cell survival. Ten isoforms have been identified within the mammalian NHE family and while the NHE1 isoform is the “housekeeping” isophorm of the exchanger, ubiquitously expressed in the plasma membrane of virtually all tissues, NHE3 is expressed at high levels in the kidney apical membranes from proximal tubule and in the mesangial cells. The hyperglycemic state increases the activity of NHEs and the plasma membrane expression of SGLT2 in a protein kinase A-dependent way, whereas hyperinsulinemia is responsible for the increased expression and activity of NHE1 and NHE3 in the glomerular mesangium, tubular epithelium, and vascular endothelium. It has been shown that increased activity of NHE3 could contribute to the onset of glomerular hyperfiltration and mesangial proliferation, while the upregulation of NHEs could lead to abnormal kidney cell growth and dysfunction [130,131,132,133,134,135,136,137].

In this context, it has been discovered that SGLT2 inhibition may indirectly lead to decreased activity of both NHE1 and NHE3 and it may inhibit NHE3 activity directly by binding to the enzyme and promoting phosphorylation of two specific serine sites, resulting in natriuresis. These agents reduce cytoplasmic sodium and calcium while increasing mitochondrial calcium levels, which may further contribute to nephroprotection [138,139,140].

#### 2.3.2. Reduction of Endothelial Dysfunction and Oxidative Stress

Endothelial dysfunction is represented by a series of modifications comprising impaired function through endothelium-dependent vasodilation and endothelial activation. The former consists of a proinflammatory, procoagulant, and proliferative state which highly favors the generation of atheroma plaques. Endothelial dysfunction appears due to the reduction of bioavailable vasodilators, of which nitric oxide (NO) is a main player. The lack of proper endothelial function has been shown to be present in persons suffering from DM. The basis for this process is that hyperglycemic states determine the depletion of NADPH, promoting reactive oxygen species (ROS) formation. Chronically high glucose concentrations generate advanced glycation end products, modifying protein structure and function and the NO synthase’s capacity to properly generate NO. Moreover, similar changes have been found in patients with moderate and end-stage kidney disease. The glomerular endothelium is the first to be affected by hyperfiltration, a condition which appears due to both mechanical factors such as increased glomerular pressure, and chemical factors such as higher glucose, nitrogen compounds, and modified ion concentrations, while hyperglycemia inhibits glycoprotein synthesis, increases apoptosis and limits the proliferation of endothelial cells [141,142].

In contrast, the treatment with SGLT2i has been shown to contribute to the up-regulation of genes involved in NO synthesis and, therefore, to increase NO bioavailability, alleviating endothelial dysfunction and preventing vascular aging, and also to reduce the levels of endothelial inflammation and oxidative stress that decrease the production of ROS. Moreover, these novel molecules contribute to vascular aging prevention via the inhibition of the proliferation and migration of smooth muscle cells in blood vessels and they have been found to offer protection from the proapoptotic effects of high glucose concentrations in proximal tubular kidney cells. Additionally, SGLT2i have been shown to inhibit monocyte chemoattractant protein-1 (MCP-1) gene expression, blocking an important factor leading to inflammation, fibrosis, and tubular atrophy in DKD [143,144,145].

#### 2.3.3. Reduction of Renal Hypoxia

Renal hypoxia is one of the main mechanisms that contributes to the development of DKD, both in its early and late phases. The proximal convoluted tubule has an elevated number of SGLT2 and, subsequently, an elevated activity of these transporters that will cause a higher adenosine triphosphate (ATP) utilization by the Na^+^/K^+^-ATPase with consecutively increased oxygen consumption. In conditions of hyperglycemia, SGLT2 activity increases through up-regulation, with an increase in oxygen consumption and renal hypoxia. Moreover, the progressive loss of function in sclerotic glomeruli damages glomerular capillaries inhibits the flow of oxygen and encourages the formation of a hypoxic microenvironment in the kidneys, with the suppression of ATP production. Renal hypoxia is augmented also by the decreased renal capacity to stimulate local renal perfusion as a consequence of microvasculature damage and lower GFR levels [146,147,148,149]

In this context, the treatment with SGLT2i contributes to the reduction of the transporter activity in the proximal convoluted tubule and, therefore, the renal oxygen consumption, resolving the intrarenal imbalance between the demand and supply of oxygen. Additionally, multiple studies have demonstrated that both acute and long-term SGLT2 inhibition lowers tubular expressions of hypoxia-inducible factor 1-alpha (HIF-1α), represses active oxygen use in the renal cortex, and decreases hyperfiltration, while through a complex mechanism, it modulates the tubular response to hypoxia by reducing high-glucose-induced O-linked N-acetylamino-glucose glycosylation modifications via the HIF pathway and, thus, it is suggested that HIF-1α inhibition is involved in renal protection. Additional benefits of SGLT2i have been shown in reducing kidney injury molecule 1 (KIM-1), a tubular injury biomarker induced by hypoxia, while similar effects have been shown in other biomarkers such as tissue necrosis factor receptor 1 (TNFR-1) and TNFR-2. Nevertheless, future studies are needed in order to gain more information regarding the unanswered questions about this process [76,150,151,152,153,154].

#### 2.3.4. Improvement of Mitochondrial Dysfunction

Mitochondria provide for cellular energy demands via the formation of ATP by maintaining a gradient potential across their inner membrane. Mitochondrial health is ensured by processes of fusion and fission and an alteration of these processes can lead to dysfunctional bioenergetics, having various implications in the development of disease. In patients with DM and poor glycemic control, high concentrations of glucose determine increased phosphorylation that will lead to an overproduction of ATP and ROS and to the oxidation of mitochondrial proteins in renal tubular cells. These changes will induce mitochondrial damage and will account for cell injury leading to the progression of diabetic nephropathy [155,156].

SGLT2i have been shown to promote ketone body production. Recent studies have shown that also the kidneys play a role in ketone production although they are a net ketone consumer. Ketone bodies have been likened to several beneficial effects: β-hydroxybutyrate reduces inflammation, atherosclerosis, and lipolysis, promotes neuroprotection, and increases reverse cholesterol transport. Moreover, it reduces ROS production, increases antioxidant levels, and improves ATP production via mitochondrial respiration. The use of ketones as an energy substrate promotes a more efficient use of oxygen, providing more ATP per 2 carbon moieties than glucose. In addition, blocking sodium reabsorption with SGLT2i reduces kidney oxygen consumption, attenuating kidney hypoxia. What is more, the glycosuric effect creates “nutrient deprivation” signaling that upregulates the effects of adenosine monophosphate-activated protein kinase (AMPK), sirtuin 1 (SIRT1) and HIF-2α, while suppressing HIF-1α. The action of the latter two mechanisms inhibits essential pathways that lead to diabetic kidney injury and consecutively contributes to renoprotection for these patients [149,157,158,159].

#### 2.3.5. Improvement of Podocyte Loss and Reduction of Albuminuria

DKD is characterized by renal hyperfiltration, glomerular basement membrane thickening, and glomerulosclerosis; however, early in the evolution of this complication, an injury followed by an important decrease in the number of podocytes can be found, with consequences in the glomerular permeability. Generally, podocytes prevent the leaking of albumin into the glomerular filtrate. In patients with DM, the increased urinary albumin excretion (UAE) is generated by alterations in the glomerular filtration barrier, and it has been shown that a lower podocyte number, the podocyte detachment, and the loss of heparan sulfate correlate directly with the UAE rate. These types of podocyte injuries can occur after an accumulation of toxic lipid metabolites.

Other mechanisms that contribute to the development of microalbuminuria in patients with DM is endothelial dysfunction, through an impaired permeability of the glomerular filtration barrier, while the tubulointerstitial injury leads to lower tubular reabsorption of filtered albumin and its dysfunctional degradation in the tubules. Nevertheless, albuminuria is considered to be not only a marker of DKD but also a cause of the disease itself [160,161,162,163,164,165,166].

In this context, SGLT2i treatment reduces the podocyte loss by causing a shift from glucose to fatty acid oxidation and, therefore, could decrease podocyte lipid content, with subsequent podocyte improved function and reduced albuminuria. Moreover, these pharmacologic agents, with their increased urinary excretion of sodium, with an increased afflux to the macula densa and subsequent afferent arterioles vasoconstriction and lowering of the intraglomerular pressure, contribute to an additional decrease in albuminuria and decreased renal disease progression [50,52,167,168,169].

#### 2.3.6. Reduction of Renal Fibrosis and Inflammation

Renal fibrosis is a common pathway and pathological marker of every type of chronic renal disease, including DKD. One of the main pro-fibrotic factors involved in this process is transforming growth factor 1 (TGF-1), which, alongside other pro-fibrotic factors such as HIF-1α and nuclear factor kappa B (NFkB), intensifies inflammation and promotes mesangial cell growth and extra-mesenchymal matrix deposition, with results of interstitial fibrosis and glomerulosclerosis. Inflammation itself plays a key role in the development of DKD: inflammatory biomarkers and growth factors such as tumor necrosis factor-alpha (TNF-α), interleukins (IL-1, IL-1, IL-16, IL-18), MCP-1 or matrix metalloproteinase-9 (MMP-9) have been found in renal biopsy samples of DM patients, while the combined action of increased insulinemia and uric acid levels, frequently encountered in these individuals, increase the mRNA expression of IL-6, IL-8 and IL-1β [170,171,172,173].

Multiple studies have shown that renal fibrosis is ameliorated by SGLT2i mainly through the reduction of hypoxia, inflammation, oxidative stress, and RAAS activation, all associated with the kidney fibrosis process. SGLT2i are able to suppress the TGF-1-related inflammatory cascade in the injured proximal tubular cells and to reduce the HIF-1α and NFkB expression, suggesting that their anti-fibrotic effects might be in direct connection with the reduced hypoxia. Moreover, SGLT2i have been shown to decrease the circulating levels of IL-6, tumor necrosis factor receptor-1 (TNF receptor-1), matrix metalloproteinase-7, and fibronectin-1, all acting on essential key points in diabetic kidney disease. The use of SGLT2i in patients with T2DM also leads to decreased levels of IL-6, while lowering uric acid and fasting insulin, and, thus, decreasing inflammation both in a direct and indirect manner. Other putative mechanisms through which this class of pharmacologic agents may be beneficial in reducing inflammation include the lowering of NFkB and profibrotic factors [120,172,173,174,175,176,177,178,179,180,181].

#### 2.3.7. Improvement of Erythropoiesis

The renal hypoxia that can be encountered in patients with DM with poor glycemic control has been shown to contribute to impaired production of erythropoietin with consecutive anemia, which appears earlier in patients with DKD than in cases of CKD or other etiologies. These mechanisms appear to be counteracted by SGLT2i which, through a decrease in renal hypoxia and subsequent reduced oxygen tension in the renal medulla, trigger a HIF-2α release, which in turn increases the synthesis of erythropoietin and erythropoiesis. Additionally, by conserving the energy needed to reabsorb the filtered glucose and associated sodium, renal hypoxia may be attenuated and is, at the same time, associated with a rise in the hematocrit [151,182,183,184,185,186,187].

#### 2.3.8. Reduction of Tubular Senescence

In the initial stages of DM, the increase in the SGLT2 activity in the proximal tubule is followed by early tubular hypertrophy and an increased glucose and sodium reabsorption with a subsequent decreased level of sodium in the macula densa, hyperfiltration, and glomerular hypertrophy. Additionally, with the reduction of sodium in the macula densa, local activation of the RAAS system will further increase the intraglomerular pressure, while angiotensin II will contribute to the progression of glomerular and tubular hypertrophy, both involved in the progression of DKD. Other factors that are involved in tubular hypertrophy are hyperinsulinemia and growth factors such as insulin-like growth factor 1 (IGF1), both related to DM, while this process involves tubular cell proliferation, cell hypertrophy and the development of a senescence-like cellular phenotype with increased secretion of pro-inflammatory cytokines, increased production of growth factors and extracellular matrix and resistance to apoptotic remodeling. Moreover, glomerular hyperfiltration, glomerular hypertrophy, and increased intraglomerular pressure will lead to glomerulosclerosis [127,188,189,190,191,192,193,194].

These effects are, as expected, counteracted by SGLT2i, since the whole pathological pathway is generated by the increase in the SGLT2 activity and also the additional effects of this pharmacological class such as the reduction in oxidative stress and even DNA damage, even to the extent in which they are considered to target aging itself, postpone age-related disease, and promote longevity [195,196].

#### 2.3.9. Improvement of Renal Autophagy and Reduction of Glomerular Damage

Autophagy is a biological process that eliminates unnecessary or damaged proteins and organelles using lysosomal vesicles called autophagosomes, essential in order to maintain cellular homeostasis. Recent studies have shown that patients with DM develop autophagy disorders caused by metabolic disruptions, altered nutrient-sensing pathways, and excessive cellular stressors, which, in turn, cause an accumulation of damaged proteins and organelles in the renal cells, with accelerated development of DKD [197,198,199].

The SGLT2i appear to have beneficial effects on the autophagy and glomerular injury process and there are several possible biochemical connections that have been suggested in this direction. Although there is still a lack of evidence, it appears that SGLT2i promote an autophagic flux in proximal renal tubule cells that eliminates intracellular waste and removes damaged organelles, which enhance tissue efficiency and cell survival. Although the exact mechanism is still unknown, SGLT2i seem to regulate autophagy and reduce DKD progression through molecular pathways that primarily involve the AMPK and mechanistic target of rapamycin (mTOR) signaling pathways, further reducing tubular and glomerular damage [140,200,201,202,203,204].

## 3. Clinical Trials and Guideline Recommendations

These renoprotective effects of SGLT2i have been shown and confirmed by a multitude of clinical trials, starting with the CV outcome trials (CVOTs) and continuing with the renal outcome trials and real-life studies. The beneficial effects of this class of pharmacological agents have been discovered during the CVOTs, which had as a main goal the assessment of CV safety. Although EMPA-REG OUTCOME (with empagliflozin in patients with T2DM with established CVD), CANVAS program (with canagliflozin in patients with T2DM and elevated risk of CVD), DECLARE-TIMI 58 (with dapagliflozin in patients with T2DM and elevated risk of CVD), and VERTIS CV (with ertugliflozin in patients with T2DM with established CVD) had primary CV outcomes, they also included “hard” renal endpoints such as the development of a composite of end-stage kidney disease (ESKD) or renal death or additional secondary outcomes such as albuminuria or other markers of kidney injury. These studies have shown that the treatment with SGLT2i could contribute to the prevention of DKD or, if already present, to delay its progression, independently from the glycemic control or the presence of CVD and heart failure (HF) [31,32,33,156,205].

Further favorable results have been encountered in DAPA-CKD and EMPA-KIDNEY, trials that assessed the impact of dapagliflozin and, respectively, empagliflozin on renal outcomes in patients with CKD, with or without DM. In these landmark trials, the reduction of the risk of a composite renal outcome—a composite of a sustained reduction of GFR of at least 50%, ESKD, or renal or CV death in DAPA-CKD or a composite of the progression of renal dysfunction or CV death in EMPA-KIDNEY—has been shown in patients with CKD, regardless of its etiology, when being treated with dapagliflozin or empagliflozin [30,62].

Moreover, a meta-analysis that included 13 trials with more than 90,000 participants from which 82.7% had DM has shown that SGLT2i reduce the risk of kidney disease progression by 37%, with similar effects in patients with DKD or other causes of CKD, while also demonstrating a statistically significant reduction in the risk of CV death [206]. Another meta-analysis that has summarized the main outcomes of SGLT2i trials, specifically, 13 trials with 90,409 participants from which 82.7% were with diabetes and 17.3% without, with a wide mean baseline eGFR range of 37–85 mL/min/1.73 m^2^, has shown their beneficial effects in slowing the renal disease progression, in lowering the risk of developing an acute kidney injury (AKI), and in decreasing the risk of a combined endpoint of cardiovascular death and hospitalization for heart failure, which are results that support their usage not only in patients with T2DM but also in those with CKD or HF, irrespective of their diabetes status, primary kidney disease, or renal function [206,207]. Additionally, a meta-analysis that has included 10 trials with more than 68,000 patients has shown that SGLT2i reduce the risk of cardiovascular mortality, hospitalization for heart failure, renal composite outcomes, and all-cause mortality, with similar results in-between molecules, depending on the selected outcome. In this study, compared to the placebo groups, canagliflozin, ertugliflozin, and sotagliflozin (a dual SGLT-2 and 1 inhibitor) have been associated with a reduction in all-cause mortality; canagliflozin, dapagliflozin, empagliflozin, ertugliflozin, and sotagliflozin with a reduction in cardiovascular mortality; canagliflozin, dapagliflozin, empagliflozin, ertugliflozin, and sotagliflozin with a reduction in hospitalizations for heart failure; and dapagliflozin, empagliflozin, canagliflozin, sotagliflozin, and ertugliflozin with a reduction in the renal composite outcome [208].

Real-life studies, complementary to the randomized controlled trials, have also confirmed these renoprotective outcomes with results that offer valuable insights into the benefits that could be expected in patients found in everyday clinical practice since it is well-known that randomized clinical trials, with their restrictive inclusion and exclusion criteria, can limit the applicability of their findings. One of the key trials, CVD-REAL3, which included more than 65,000 patients, has shown a 51% reduction of a GFR decrease of more than 50% or the development of ESKD in patients treated with SGLT2i, results obtained in the entire GFR and albuminuria spectrum, with similar results found in multiple other real-life studies that have also suggested beneficial effects in AKIs [209,210,211].

The renoprotective effects of SGLT2i have been proven in patients with and without DM, regardless of the CKD etiology, as previously mentioned, not only in trials such as DAPA-CKD and EMPA-KIDNEY but also in multiple other studies. In non-DM patients, the renoprotection of SGLT2i is independent of their effects on blood glucose levels. If in patients with DM, the alteration of the tubuloglomerular feedback mechanism is the main cause of CKD progression; with decreased sodium delivery to the macula densa and consecutive vasodilation of the afferent arteriole and increased intraglomerular pressure, the CKD in patients without DM appears to have similarities with these mechanisms, with alterations in the tubuloglomerular feedback and glomerular perfusion autoregulation being also described in these cases. As already shown, SGLT2i restore the tubuloglomerular feedback, while also reducing the levels of proinflammatory mediators such as IL-6, TNF, and C-reactive protein and also reducing the quantity of perirenal fat and its characteristics, lowering its proinflammatory and fibrogenic capacity [30,62,212,213,214,215,216,217].

An established therapy for patients with CKD, the RAAS inhibitors, including the angiotensin-converting enzyme (ACE) inhibitors and angiotensin receptor blockers (ARBs), are considered major therapeutic advances since their strong evidence from large randomized controlled trials that have shown their benefits in lowering blood pressure, preventing target organ damage in hypertension, reducing mortality in heart failure and the aforementioned effect in lowering proteinuria and slowing the progressive loss of kidney function in patients with renal disease. These RAAS blockers are proven to be the most effective in slowing CKD progression when prescribed in the early stages; however, their benefits in advanced CKD are currently being debated [218,219,220,221].

**Figure 5:**
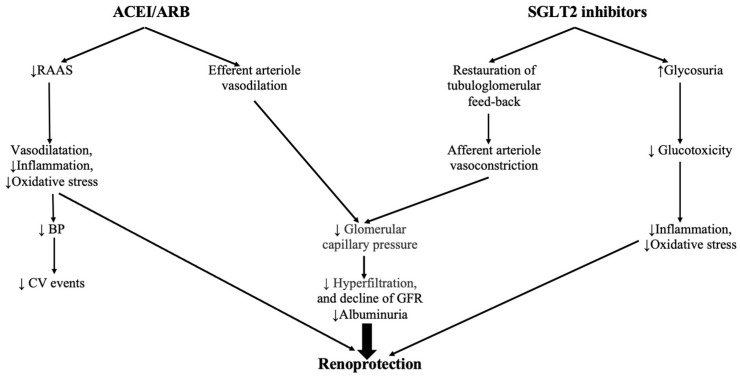
The renoprotective effects of angiotensin-converting enzyme (ACE) inhibitors/angiotensin receptor blockers (ARBs) and SGLT2i (Adapted from Fujimoto et al.) [220]. ↑ increase, ↓ decrease.

Nevertheless, RAAS inhibition is associated in multiple studies with a lower incidence of the progression to end-stage renal disease in DKD patients and a lower incidence of composite MACE, being an important therapeutic option in DKD patients with albuminuria and glomerular disease. The RAAS cascade contributes to the increased production of angiotensin II (Ang II) which, by binding to the Ang II type 1 receptor, leads to vasoconstriction, cell proliferation, inflammation, increased oxidative stress, and cell apoptosis and, thus, its inhibition blocks this pathological pathway and leads to vasodilation of the efferent arteriole with a decrease in the filtration pressure. In this context, it has been shown that the ACEI/ARBs and the SGLT2i play different roles in different sites of the nephron and have a potentially synergistic mechanism of action in patients with CKD, with the SGLT2i reversing the afferent arteriole vasodilation, while the RAAS blockers induce the vasodilation in the efferent arteriole, with subsequent decrease of the glomerular pressure and, thus, with their combination being associated with a higher renoprotection than the administration of either agent in monotherapy [222,223,224,225,226]. These pathways through which both SGLT2i and ACEI/ARBs are protecting the renal function are presented in Figure 5.

Regarding their safety, the most frequently encountered adverse events associated with the usage of SGLT2i are genital infections—with a 2–6 fold increased risk compared to placebo, depending on the trial—that, in some patients, can lead to treatment discontinuation or to a negative effect on the quality of life. Nevertheless, the majority of these cases are mild and respond to standard treatment with topical or single-dose oral antifungals [206,227,228,229,230]. Regarding other adverse effects, a meta-analysis that included more than 78,000 participants of which some with T2DM, heart failure, high risk of atherosclerotic CVD, or manifest CVD or CKD, has shown superiority of SGLT2i vs. placebo in reducing the risk of AKI, while increasing the risk of diabetic ketoacidosis—with possible euglycemic ketoacidosis, a rare adverse effect, possibly found in 1 in 1000 SGLT2i users, although its incidence is yet uncertain—and volume depletion, situations that can be avoided by stopping their administration in prolonged fasting, surgery or critical illness. However, the treatment with SGLT2i did not show any significant effects on the incidence of urinary tract infections—contrary to previous beliefs—or on amputations, bone fractures, thromboembolic events, or hypoglycemia [231,232,233].

When analyzing the differences in the effectiveness and safety of various SGLT2i, the effects in reducing CV and kidney outcomes and the safety profiles among molecules did not show consistent statistical significance, regardless of the presence of DM, while for adverse events, empagliflozin, and dapagliflozin have proven a lower risk of developing AKI, while also significantly reducing the rate of the kidney disease progression [234].

These trials have led to considering SGLT2i as being disease-modifying drugs, not only in patients with DM or HF but also with CKD, regardless of the glycemic control, cause of CKD, GFR, or albuminuria. In this context, recent guidelines have included SGLT2i as a first-line therapy for the prevention or delayed progression of renal disease. The American Diabetes Association 2024 Standards of Care recommend, for the management of patients with T2DM and CKD, to preferably administer an SGLT2i with primary evidence of reducing CKD progression that can be initiated when the GFR ≥ 20 mL/min/1.73 m^2^ and, once initiated, it should be continued until the initiation of dialysis or transplantation. Similarly, in the 2023 European Society of Cardiology Guidelines for the management of cardiovascular disease in patients with diabetes, it is recommended to initiate the therapy with SGLT2i in patients with T2D to reduce cardiovascular and kidney failure risk, while the 2022 KDIGO Clinical Practice Guideline for Diabetes Management in Chronic Kidney Disease and the 2024 KDIGO Clinical Practice Guideline for the Evaluation and Management of Chronic Kidney Disease recommend, as first-line therapy, the usage of SGLT2i in all patients with T2D and CKD or in patients with CKD, without DM, with either an albumin/creatinine ratio ≥20 mg/mmol (200 mg/g), or HF, while this usage is suggested in patients with a GFR 20–45 mL/min/1.73 m^2^ and an albumin/creatinine ratio <20 mg/mmol (200 mg/g) [20,231,235,236,237].

## 4. Conclusions

With all the previously mentioned mechanisms, it becomes evident that after decades in which no significant progress regarding the prevention and delayed decline of renal function in patients with CKD, with or without DM, has been made, the development of SGLT2i has brought a significant change in the approach of these individuals, being considered the most important step forward in renoprotection since the discovery of RAAS inhibitors. With each metabolic, hemodynamic, and direct renal effect, SGLT2i reduce the risk of CKD occurrence and progression, both in people with DM and in those without, regardless of the cause of the kidney disease, through multiple pathways, many of them independent of glucose reduction, values of blood pressure or body weight and many of them still unknown or incompletely elucidated. Nevertheless, the ongoing discovery of the complex mechanisms that contribute to the development of renal dysfunction and the continuous research regarding innovative pharmacologic agents contribute significantly to attaining the ideal outcome of renal prevention and to a new dawn in the management of patients with CKD.

## Figures and Tables

**Table 1:** Nephroprotective mechanisms of SGLT2i.

Category	Nephroprotective Effects of SGLT2i
Metabolic effects	Reduction in plasma glucose levels Reduction of glucotoxicity Reduction of body weight Reduction of lipotoxicity Reduction of triglycerides and small-dense LDLc Improvement of insulin sensitivity Reduction of hepatic steatosis Reduction of plasma uric acid levels Metabolic reprogramming (restoration of energy efficiency by switching glucose to more energy efficient metabolites)
Hemodynamic effects	Natriuresis Improvement of salt-sensitive hypertension Reduction of blood pressure Improvement of sympathetic nerve hyperactivity Reduction of volume overload Modulation of the tubuloglomerular feedback
Direct nephroprotective effects	Inhibition of the Na-H exchanger Reduction of endothelial dysfunction and oxidative stress Reduction of renal hypoxia Improvement of mitochondrial dysfunction Improvement of podocyte loss Reduction of albuminuria Reduction of renal fibrosis Reduction of inflammation Improvement of erythropoiesis Reduction of tubular senescence Improvement of renal autophagy Reduction of glomerular damage
